# Long-term hospital admissions and surgical treatment of children with congenital abdominal wall defects: a population-based study

**DOI:** 10.1007/s00431-021-04005-2

**Published:** 2021-03-05

**Authors:** Arimatias Raitio, Johanna Syvänen, Asta Tauriainen, Anna Hyvärinen, Ulla Sankilampi, Mika Gissler, Ilkka Helenius

**Affiliations:** 1 Department of Paediatric Surgery, University of Turku and Turku University Hospital, Kiinamyllynkatu 4-8, 20521 Turku, Finland; 2 University of Eastern Finland, Kuopio, Finland; 3 Department of Paediatric Surgery, University of Tampere and Tampere University Hospital, Tampere, Finland; 4 Department of Paediatrics, Kuopio University Hospital, Kuopio, Finland; 5 Information Services Department, Finnish Institute for Health and Welfare, Helsinki, Finland; 6 Department of Neurobiology, Care Sciences and Society, Karolinska Institute, Solna, Sweden; 7 Department of Orthopaedics and Traumatology, University of Helsinki and Helsinki University Hospital, Helsinki, Finland

**Keywords:** Congenital abdominal wall defect, Exomphalos, Gastroschisis, Omphalocele, Hospital care

## Abstract

Congenital abdominal wall defects, namely, gastroschisis and omphalocele, are rare congenital malformations with significant morbidity. The long-term burden of these anomalies to families and health care providers has not previously been assessed. We aimed to determine the need for hospital admissions and the requirement for surgery after initial admission at birth. For our analyses, we identified all infants with either gastroschisis (*n*=178) or omphalocele (*n*=150) born between Jan 1, 1998, and Dec 31, 2014, in the Register of Congenital Malformations. The data on all hospital admissions and operations performed were acquired from the Finnish Hospital Discharge Register between Jan 1, 1998, and Dec 31, 2015, and compared to data on the whole Finnish pediatric population (0.9 million) live born 1993−2008. Patients with gastroschisis and particularly those with omphalocele required hospital admissions 1.8 to 5.7 times more than the general pediatric population (*p*<0.0001). Surgical interventions were more common among omphalocele than gastroschisis patients (*p*=0.013). At the mean follow-up of 8.9 (range 1.0–18.0) years, 29% (51/178) of gastroschisis and 30% (45/150) of omphalocele patients required further abdominal surgery after discharge from the neonatal admission.

*Conclusion*: Patients with gastroschisis and especially those with omphalocele, are significantly more likely than the general pediatric population to require hospital care. Nevertheless, almost half of the patients can be treated without further surgery, and redo abdominal surgery is only required in a third of these children.

**Table:** 

**What is Known:** • *Gastroschisis and omphalocele are congenital malformations with significant morbidity* • *There are no reports on the long-term need for hospital admissions and surgery in these children* **What is New:** • *Patients with abdominal wall defects are significantly more likely than the general pediatric population to require hospital care* • *Almost half of the patients can be treated without further surgery, and abdominal redo operations are only required in a third of these children*

**What is Known:**

• *Gastroschisis and omphalocele are congenital malformations with significant morbidity*

• *There are no reports on the long-term need for hospital admissions and surgery in these children*

**What is New:**

• *Patients with abdominal wall defects are significantly more likely than the general pediatric population to require hospital care*

• *Almost half of the patients can be treated without further surgery, and abdominal redo operations are only required in a third of these children*

## Introduction

Major congenital anomalies, including gastroschisis and omphalocele, have an impact on the quality and length of life of affected individuals. Both these aforementioned abdominal wall defects (AWDs) are relatively rare congenital anomalies with respective prevalences of 1.85 and 1.96 per 10,000 births in Finland [1, 2]. Gastroschisis often presents as an isolated anomaly [1, 3] and has good long-term outcomes, and abdominal reoperations are rarely required [4]. Omphalocele, on the other hand, is often associated with other severe comorbidities including chromosomal abnormalities and cardiac defects [2, 5, 6]. Consequently, up to 60% of patients with giant omphalocele suffer from persistent medical problems [7, 8]. However, long-term complaints are rare among patients with small omphalocele [9].

There are a handful of studies on the burden imposed by selected congenital anomalies and Down’s syndrome on hospital care [10–14]. The data on the need, duration, and frequency of hospital admissions and surgical treatment are important not only for health care providers but also for families and caregivers preparing to live with a child with a congenital anomaly. Early studies have demonstrated that gastroschisis is associated with significant morbidity and even mortality after the neonatal period [15], especially among patients with complex gastroschisis [16–18]. However, there are so far no published studies on the long-term need for hospital admission and operations among patients with gastroschisis and particularly omphalocele during childhood.

The purpose of this population-based register study was therefore to assess the burden associated with AWDs on patients’ families and on the health care system by determining the number of hospital admissions, total time spent in hospital, and the number of surgical interventions in children with gastroschisis and omphalocele. Additionally, we wanted to compare these data with those of the general pediatric population of our country. We hypothesized that children with gastroschisis would have significantly less need for hospital readmissions and redo abdominal surgery after initial admission than children with omphalocele.

## Material and methods

All children born with AWD between Jan 1, 1998, and Dec 31, 2014, were identified in the Finnish Register of Congenital Malformations (FRM) allowing a minimum of one-year follow-up. The register contains data on all live births, stillbirths, and fetuses from spontaneous abortions and elective terminations of pregnancy for fetal anomalies, all with at least one major congenital anomaly. These anomalies and chromosomal defects are coded according to an extended version of the 9th Revision of the International Classification of Diseases (ICD-9) of the World Health Organization. According to the system of the European Surveillance of Congenital Anomalies (EUROCAT) [19], minor anomalies were excluded. The data on hospital admissions were collected from the Finnish Hospital Discharge Register (FHDR). Both these registers are maintained by the Finnish Institute for Health and Welfare (THL).

FRM receives nationwide data on congenital and fetal anomalies from hospitals, healthcare professionals, and cytogenic laboratories. With the help of the unique personal identification code (PIC), FRM also draws data from other national health registers including Medical Birth Register, Register on Induced Abortions, FHDR, and The Register of Visual Impairment, all maintained by THL, as well as from Cause-of-Death Register, maintained by Statistics Finland. The data quality and coverage of these registers have been validated and considered good in several studies [20–23].

Nationwide data on all hospital discharges and outpatient visits are registered in FHDR, and the study population identified in FRM was cross-linked with the FHDR data by the PIC. Basic variables collected in FHDR include the date of birth, sex, area of residence, admission and discharge dates, surgical procedures, and diagnoses of patients’ medical problems. Diagnoses were recorded according to the ICD-10, and the operations were registered according to the Finnish version of NOMESCO (Nordic Medico-Statistical Committee) Classification of Surgical Procedures (NCSP). Numbers of all hospital admissions in gastroschisis and omphalocele patients (excluding the birth episode) between Jan 1, 1998, and Dec 31, 2015, were analyzed and compared with the whole live born pediatric population 1993−2008 (*n*=942,692). Surgical and nonsurgical admissions were analyzed separately including days spent in the hospital, as well as number and type of surgical operations. Surgical procedures were categorized by surgical specialty and general pediatric surgery procedures by anatomical location (abdominal, groin, and intravenous access).

### Statistical analysis

A one-sample *t* test was used to compare continuous variables, and Kaplan-Meier analysis was utilized for operation-free survival. A significance level of *p* < 0.05 (two-tailed) was set. Analyses were performed using JMP Pro, version 13.1.0 for Windows (SAS Institute Inc., Cary, North Carolina, USA).

### Ethical considerations

The approval of the Institutional Review Board at Turku University Hospital was obtained before conducting this study. The Finnish Institute for Health and Welfare gave permission to use their health register data in this study.

## Results

We identified 178 infants with gastroschisis and 150 with omphalocele in the registers born between Jan 1, 1998, and Dec 31, 2014. These 328 children with AWDs had altogether 1,507 hospital admissions and spent 7,465 days in the hospital during our 18-year study period (Table 1). The median follow-up time was 8.2 (range 1.0–18.0) years for gastroschisis and 9.9 (range 1.2–17.9) years for omphalocele patients. The whole live born pediatric population 1993−2008, used as a reference, had 1,524,481 hospital admissions in total with 4,194,675 hospital days during the 17–year period. The mean number of hospital admissions in our control population was thus 0.10, and the mean length of inpatient care is 0.3 days per child per year.

**Table 1:** The number of patients with hospital admissions in gastroschisis and omphalocele

	Patients with hospital admissions (%) Range of admissions/patient	Median days in hospital (range)
Gastroschisis (*n*=178)	143 (80.3%) 0–31	5 (0–302)
Omphalocele (*n*=150)	116 (77.3%) 0–63	6 (0–387)

In gastroschisis patients, the annual mean number of hospital admissions was 0.18 with 0.9 days on average spent in the hospital. The corresponding numbers for omphalocele were 0.34 admissions and 1.7 days in hospital annually. In gastroschisis patients, the number of admissions and inpatient days was, respectively, 1.8 and 3.0 times higher than that in the general population (*p*<0.0001 and *p*=0.003). Omphalocele patients, on the other hand, were annually 3.4 times more likely to be admitted to the hospital and spent 5.7-fold more days per year as inpatients (*p*<0.0001 for both). The majority of the admissions were nonsurgical among both gastroschisis and omphalocele patients; 71% and 64% of all admissions, respectively. The first year of life accounted for 45% of these admissions in gastroschisis and 35% in omphalocele patients.

The most common types of surgery were ear, nose, and throat (ENT) operations, gastrointestinal (GI) surgery, and groin surgery, which included operations for inguinal hernia and undescended testicles. Bowel obstruction required surgery in nine (5%) gastroschisis and four (3%) omphalocele patients. Other laparotomies in 20 gastroschisis and 18 omphalocele patients involved bowel resections, stoma formations and closures, and antireflux surgery. A significant number of operations related to intravenous (IV) access were observed among patients with omphalocele, and contrary to the gastroschisis cohort, they also required cardiac and urological procedures (Table 2).

**Table 2:** The percentage and number of patients requiring surgery after initial admission among gastroschisis and omphalocele patients

	All surgery (*n*) Range of operations/patient	GI surgery (n) Range of operations/patient	Groin surgery (*n*) Range of operations/patient	IV access (*n*) Range of operations/patient	Urologic surgery (*n*) Range of operations/patient	Orthopedic surgery (*n*) Range of operations/patient	ENT operations (*n*) Range of operations/patient	Cardiac surgery (*n*) Range of operations/patient
Gastroschisis (*n*=178)	48% (86) 0–14	16% (29) 0–4	14% (25) 0–2	8% (15) 0–4	0	2% (4) 0–2	19% (33) 0–7	0
Omphalocele (*n*=150)	62% (93) 0–31	15% (22) 0–6	25% (37) 0–4	15% (23) 0–3	6% (9) 0–15	7% (11) 0–12	23% (34) 0–9	8% (12) 0–6

**Fig. 1:**
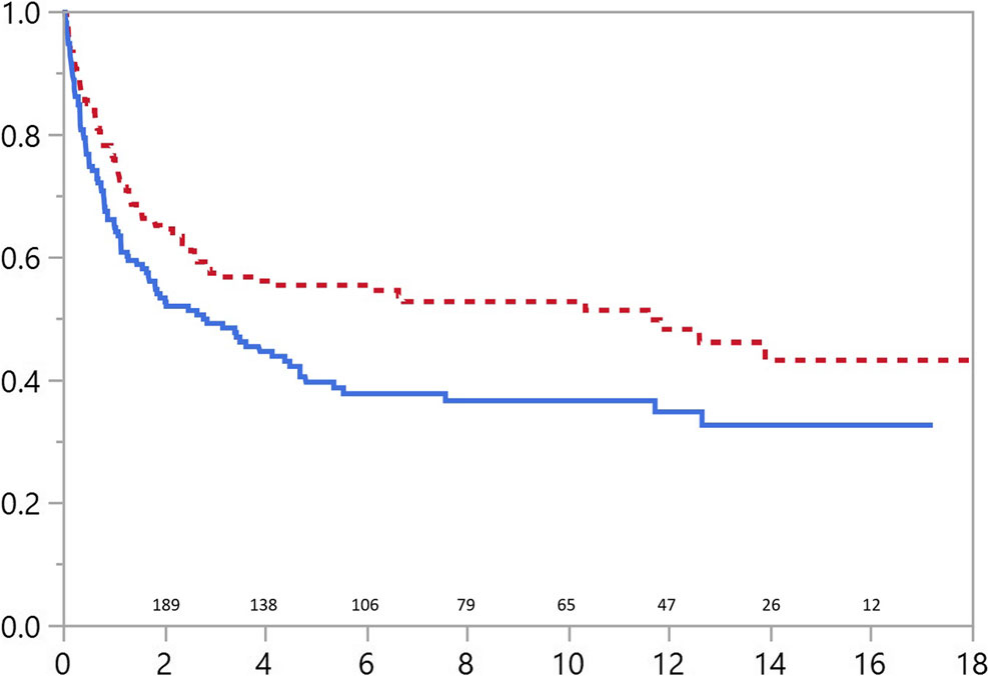
Operation-free survival for patients with abdominal wall defects. Solid line representing omphalocele and dotted line gastroschisis patients. Number of patients at risk and years of follow-up in *x* scale

**Fig. 2.**
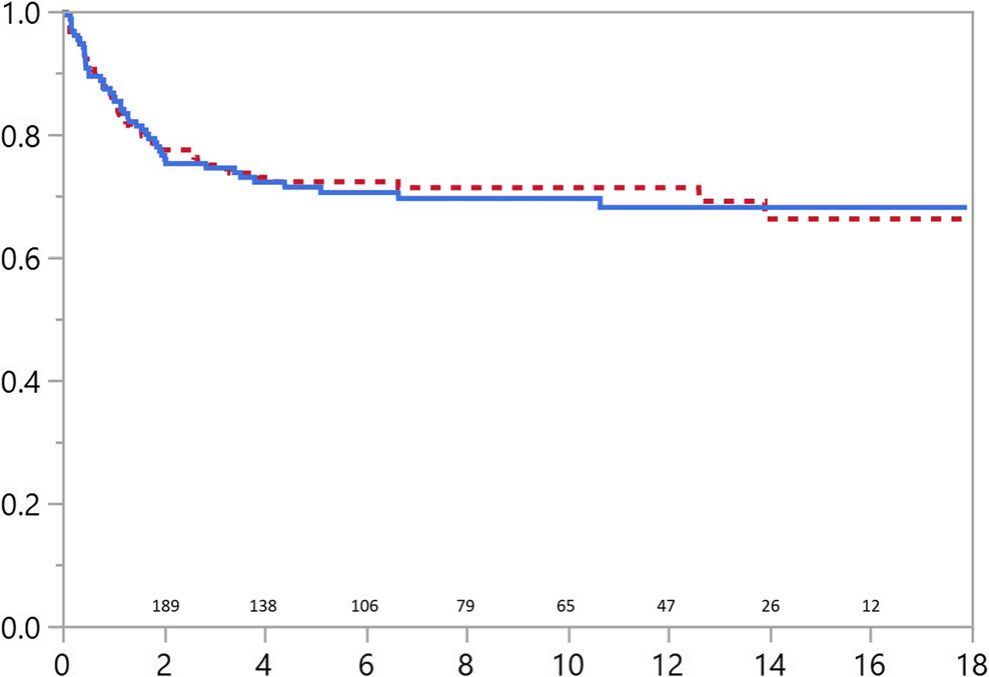
Survival without redo abdominal or abdominal wall hernia surgery in patients with abdominal wall defects. Solid line depicting omphalocele and dotted line gastroschisis patients

Patients with omphalocele were statistically more likely to require surgery than those with gastroschisis after their initial admission (*p*=0.013, Fig. 1). Gastrointestinal operations and/or abdominal wall hernia operations were equally common in both patient groups: 51/178 (29%) in gastroschisis and 45/150 (30%) in omphalocele (Fig. 2). Recurrent or residual abdominal wall hernia operations were performed in 25/178 (14%) patients with gastroschisis and 28/150 (19%) with omphalocele. As presented in Fig. 1, the majority of omphalocele patients (55%) and 48% of gastroschisis patients requiring surgery were operated on before the age of one year, and after, infancy surgical intervention was required less often.

## Discussion

According to this population-based study, the need for hospital care among patients with AWDs is multiple-fold compared to the general pediatric population. To the best of our knowledge, no studies on long-term need for care and operations among AWD patients have been published. Children with major birth defects have been reported to be 2.5 times more likely on average to require hospital care than children without congenital defects [10], and even higher numbers have been reported with orofacial clefts [13] and limb deficiencies [14].

In our study, both gastroschisis and omphalocele were associated with significantly higher frequency and duration of hospital admissions than the general pediatric population. An Australian study by Colvin et al. [10] reported childhood hospital admissions to be 2.5 times more likely among children with major congenital anomalies, which is well within the range of our findings among gastroschisis patients. Omphalocele, on the other hand, was associated with over fivefold risk of inpatient stay, which is comparable with English national hospital admission data on patients with cleft lip and/or palate [13]. We postulate that the greater number of hospital admissions in omphalocele is likely a reflection of the greater number of associated anomalies and syndromes often seen in these patients [2, 24–26].

Less than half of the patients with gastroschisis (48%) required surgical intervention after their initial admission at birth. According to the published literature, umbilical hernia appears to be rather common after gastroschisis repair [27, 28]. Sutureless closure has been reported to be associated with 13% incidence of umbilical hernia requiring surgical repair [28]. We reported a surgical repair rate of 14%, which may at least partially be explained by the longer follow-up period than in previously published studies. De Bie et al. reported a 5.6% risk of acute abdominal complications in long-term follow-up among patients with simple gastroschisis. In our cohort, 17% of patients underwent GI surgical operations including both elective and emergency procedures. However, our cohort also included patients with complex gastroschisis which is reported to be associated with over 60% risk of reoperation [29].

Majority of omphalocele patients (62%) required surgical care after being discharged. As omphalocele is often associated with other congenital anomalies, a wider range of operations including cardiac, orthopedic, and urological surgery was performed on omphalocele patients in contrast to gastroschisis. Also, operations for undescended testicles were more common among omphalocele than among gastroschisis patients as we reported earlier [30]. Adhesive bowel obstruction has previously been reported to occur in up to 15% of omphalocele patients [31, 32], which is in line with our 15% frequency of GI surgical reoperation reported here. The requirement for reoperation(s) due to abdominal wall defect depends on the size of the defect and the methods of treatment [33]. We reported a somewhat higher rate of hernia operations among omphalocele patients than in gastroschisis (19% vs. 14%). However, this is likely to include cases with initial conservative treatment of the defect with delayed fascial closure, which makes comparison with previous data challenging.

The strength of the study is that the register data stored in the FRM and the FHDR are both validated with high accuracy and full country coverage [34, 35]. All hospitals report to the register, and there are no private children’s hospitals in Finland. Before entering the data in the register, all case data were further validated by examining all available medical records and radiographs. Furthermore, hospitals are expected to report the diagnosis and operation codes accurately as these are the bases for hospital billing [36]. In addition, the data on the control group was derived from a population-based register. The weakness of this study is the shorter follow-up time in cases born recently.

In conclusion, patients with congenital AWD, especially those with omphalocele, are significantly more likely to require hospital care than the general pediatric population. However, almost half of the patients can be treated without further surgery, and redo abdominal surgery is only required in a third of these children.

## Data Availability

The data that support the findings of this study are available from the corresponding author upon reasonable request.

## Abbreviations

AWD: Abdominal wall defect
ENT: Ear, nose, and throat
FHDR: Finnish Hospital Discharge Register
FRM: Finnish Register of Congenital Malformations
GI: Gastrointestinal
ICD: International Classification of Diseases
IV: Intravenous
PIC: Personal identification code
THL: Finnish Institute for Health and Welfare

## Code availability

JMP Pro, version 13.1.0 for Windows (SAS Institute Inc., Cary, North Carolina, USA).
